# Testis developmental related gene 1 regulates the chemosensitivity of seminoma TCam‐2 cells to cisplatin via autophagy

**DOI:** 10.1111/jcmm.14654

**Published:** 2019-09-09

**Authors:** Dongyi Peng, Jingchao Wei, Yu Gan, Jianfu Yang, Xianzhen Jiang, Riko Kitazawa, Yali Xiang, Yingbo Dai, Yuxin Tang

**Affiliations:** ^1^ Department of Urology The Third Xiangya Hospital of Central South University Changsha China; ^2^ Department of Urology Xiangya Hospital of Central South University Changsha China; ^3^ Department of Diagnostic Pathology Ehime University Hospital Toon Japan; ^4^ Department of Health Management Center The Fifth Affiliated Hospital of Sun Yat‐sen University Zhuhai China; ^5^ Department of Urology The Fifth Affiliated Hospital of Sun Yat‐sen University Zhuhai China

**Keywords:** autophagy, chemosensitivity, cisplatin, testicular seminoma, testis developmental related gene 1

## Abstract

We previously identified testis developmental related gene 1 (TDRG1), a gene implicated in proliferation of TCam‐2 seminoma cells. Recent evidence has revealed that autophagy influences the chemosensitivity of cancer cells to chemotherapy. However, whether TDRG1 protein regulates autophagy in seminoma cells and influences their sensitivity to *cis*‐dichlorodiammine platinum (CDDP) remains unknown. In this study, we used TCam‐2 cells and male athymic BALB/c nude mice with xenografts of TCam‐2 cells to investigate autophagy, cell viability, apoptosis and the p110β/Rab5/Vps34 (PI3‐kinase Class III) pathway under the conditions of TDRG1 overexpression or knockdown and with or without CDDP treatment. We found that TDRG1 upregulation promoted autophagy in both TCam‐2 cells and seminoma xenografts via p110β/Rab5/Vps34 activation. Inhibition of autophagy reduced cell viability and promoted apoptosis during CDDP treatment of TCam‐2 cells. Similarly, TDRG1 knockdown inhibited autophagy, reduced cell viability and promoted apoptosis during CDDP treatment of TCam‐2 cells. TDRG1 knockdown inhibited tumour growth and promoted apoptosis in TCam‐2 cell xenografts, whereas TDRG1 overexpression had the opposite effect. According to these results, we propose that high expression of TDRG1 promotes autophagy through the p110β/Rab5/Vps34 pathway in TCam‐2 cells. TDRG1 overexpression promotes autophagy and leads to CDDP resistance, whereas TDRG1 knockdown inhibits autophagy and promotes chemosensitivity to CDDP both in vivo and in vitro. This study has uncovered a novel role of TDRG1 in reducing chemoresistance during CDDP treatment and provides potential therapeutic strategies for the treatment of human seminoma.

## 1 INTRODUCTION

Testicular germ cell tumours (TGCT) is the most common malignancy in young men aged from 15 to 40 years, although it accounts for only 2% of all male malignancies.^1^ Pure seminoma is a well‐defined clinical and pathological entity, comprising 40%‐60% of all TGCT, and its incidence been increasing worldwide for decades.^2^ For patients with early‐stage seminoma, including clinical Stage I and low‐volume Stage II seminoma, inguinal orchiectomy followed by suitable radiotherapy is usually curative.^3^, ^4^ In patients with more advanced stages, *cis*‐dichlorodiammine platinum (CDDP)‐based chemotherapy regimens can achieve complete response rates of 70%‐90%.^3^, ^5^, ^6^ However, some patients with advanced‐stage seminoma are resistant to CDDP chemotherapy, representing either refractory or relapse cases.^7^, ^8^, ^9^ Thus, novel treatments to improve the overall chemosensitivity of seminoma are urgently needed.

Autophagy is an ancient and highly conserved process in which unneeded or damaged proteins, or other cytoplasmic components are transported to the lysosomal system to degrade.^10^ As it was involved in many cellular processes, autophagy plays important roles in many kinds of diseases, including cancer.^11^, ^12^, ^13^ But autophagy is a “double‐edged sword” for cancer cells during tumour development.^14^, ^15^ Autophagy is thought to help prevent cancer initially; however, once a cancer is formed, autophagy activation may promote the growth and survival of tumour cell.^16^, ^17^ In chemotherapy, chemotherapeutic drugs kill cancer cells mainly by inducing apoptosis. However, the activation of autophagy and relevant pathways can inhibit apoptosis, which promotes resistance to chemotherapy.^18^, ^19^ Very recently, many studies have focused on inhibition of autophagy as a way to enhance chemosensitivity in mesothelioma, colorectal cancer, hepatocellular carcinoma, neuroblastoma and other kinds of cancers,^20^, ^21^, ^22^, ^23^ and have reported very good outcome. Therefore, autophagy could be a potential therapeutic target for improving the chemosensitivity of seminoma as well.

In metazoans, the initiation of autophagy is regulated by phosphoinositides, a product of phosphoinositide 3‐kinases (PI3Ks). According to sequence homology and substrate specificity, PI3K lipid kinases are divided into three classes (class I‐III).^24^, ^25^ Class III PI3K converts phosphatidylinositol into phosphatidylinositol 3‐phosphate (PI(3)P) by binding catalytic subunit Vps34 to regulatory subunit Vps15, which is essential for the initiation of autophagy.^26^, ^27^, ^28^ Recently, it was shown that the p110β (the catalytic subunit of class I PI3Ks) interacts with the small GTPase Rab5 to promote autophagy by maintaining Rab5 in the binding state of guanosine triphosphate (GTP) and improving the Rab5‐Vps34 interaction.^29^, ^30^

Testis developmental related gene 1 (TDRG1) is a new gene identified by our group which is expressed in human testis and some higher non‐human primates. Our previous study demonstrated that it encodes a 100‐amino acid protein (TDRG1 protein) whose expression is enhanced in testicular seminoma than in normal testis. Moreover, we showed that TDRG1 facilitates the invasiveness and proliferation of seminoma cells by activating the PI3K/Akt signalling pathway, meaning that TDRG1 has a carcinogenic role in seminoma.^31^

Although inhibition of autophagy can enhance chemosensitivity in many kinds of cancers, whether TDRG1 is involved in regulating the chemical sensitivity of CDDP through autophagy in seminoma remains unclear. In the present study, we investigated the effects of TDRG1 protein on tumour growth in vivo and on autophagy, cell viability, apoptosis and the p110β/Rab5/Vps34 (PI3‐kinase Class III) pathway of seminoma TCam‐2 cells in vitro, with or without CDDP treatment. Our results demonstrate that TDRG1 regulates the chemosensitivity of TCam‐2 cells to CDDP via autophagy through a Class III PI3K.

## 2 MATERIALS AND METHODS

### 2.1 Ethics statement

The Institutional Research Ethics Committee of Third Xiangya Hospital approved this study. Informed consent and all experiments were consistent with the principles of the Declaration of Helsinki, and all patients provided written‐informed consent for the use of their tissue samples and records.

### 2.2 Tissue and cell culture

Testicular seminoma tissues (n = 10) and normal testicular tissues (n = 10) were collected from the Affliated Cancer Hospital of Xiangya School of Medicine and the Third Xiangya Hospital of Central South University. Western blotting was used to check the expression of LC3‐II and TDRG1 in the two kind of tissue samples.

The human TCam‐2 cells were gifted from Dr Riko Kitazawa, Department of Diagnostic Pathology, Ehime University Hospital, Matsuyama, Japan. TCam‐2 cells were cultured in Roswell Park Memorial Institute Medium‐1640 containing 10% FBS, 1% Pen/Strep.

### 2.3 RNAi

For RNAi, two small interfering RNAs (siRNA) targeting PI3K/p110β and Rab5 were designed, synthesized and inserted into the vector of pGPU6/GFP/Neo. We choose the highest inhibition efficient siRNA: Forward primer: 5′‐AAUCCCUCUAAAUAUCAGATT‐3′; Reverse primer: 5′‐UCUGAUAUUUAGAGGGAUUTT‐3′ for PI3K/p110β, Forward primer: 5′‐GCAGCCUUCCUUUCCAAAGTT‐3′; Reverse primer: 5′‐AACUUUGGAAAGGAAGGCUTT‐3′ for Rab‐5, and an siRNA control vector (Forward primer: 5′‐UUCUCCGAACGUGUCACGUTT‐3′; Reverse primer: 5′‐ACGUGACACGUUCGGAGAATT‐3′) was also used (Invitrogen Life Technologies).

After cells reaching 70% confluence, the above vectors were transfected into TCam‐2 cells by Lipofectamine 2000 (Invitrogen Life Technologies) following the instructions. GFP protein expressed by cells were imprinted using a laser confocal scanning microscope (Leica tcs‐sp5) to check transfection efficiency. Meanwhile, the expression of PI3K/p110β and Rab5 was checked by Western blot.

### 2.4 Autophagy flux assay

After different treatment, the autophagic flux in TCam‐2 cells was determined by quantifying LC3‐II with Western blot or immunofluorescence imaging. Briefly, TCam‐2 cells were exposed to Baf A1 (Bafilomycin A1) in the last 6 hours before harvesting. After incubated in lysosomal inhibitor Baf A1 for 6 hours, the autophagy protein LC3‐II accumulated and the amount depending on the autophagic flux.

### 2.5 Vps34 kinase assay

Class III PI3K ELISA Kit (Echelon, Lot# K 3000) was used to check the Vps34 (PI3‐Kinase Class III) activity. Firstly, the Vps34 enzyme was reprecipitated by anti‐hvps34 antibody from TCam‐2 cells, then added 20 µL kinase reaction buffer, 4 µL of 500 µmol/L phosphatidylinositol (PtdIns) substrate and 1 µL of 1.25 mmol/L ATP and incubated for 1 hour at room temperature. Then quenched the mixture was with secondary Detection Buffer (5 µL of 100 mmol/L EDTA, diluted with 130 µL H_2_O and 40 µL PI(3)P Detection Buffer) and incubated for 30 minutes at room temperature. Finally, TMB Substrate was added to the quenched reaction mixture and incubated at room temperature for 30 minutes in dark and then added Stop Solution. Read the absorbance at 450 nm on a plate reader.

### 2.6 Rab5 activity assay

BL21‐CodonPlus *Escherichia coli* was transformed with pGEX‐4 T‐2/Rabaptin‐5:R5BD and then induced with 0.2 mmol/L IPTG at 28°C for 4 hours. Then made the incubation buffer freshly containing 20 mmol/L HEPES, 100 mmol/L NaCl, 1 mmol/L DTT, 1 mmol/L GTPγS and 5 mmol/L MgCl_2_, and adjust the pH to 7.5. Then loaded bacterial lysates to glutathione agarose beads (Invitrogen). After wash, incubated with incubation buffer at room temperature for 90 minutes. Then, we get the GST‐R5BD which was stabilized at room temperature for another 20 minutes. For pull‐down assay, TCam‐2 cells in 10‐cm plates were lysed in lysis buffer containing 1 mmol/L DTT, 100 mmol/L NaCl, 1 mmol/L CaCl_2_, 25 mmol/L HEPES, 5 mmol/L MgCl_2_, 10% glycerol, 100 μmol/L PMSF, 1% NP‐40, pH 7.4 and EDTA‐free protease inhibitor cocktail. After centrifugation in 4°C for 10 minutes, the supernatants were isolated and incubated with GST‐R5BD beads at 4°C for another 10 minutes, then boiled in 1× SDS sample buffer and subjected to Western blot. At the same time, the total Rab5 protein was also determined by Western blot.

### 2.7 Cell viability assay

The MTT assay was performed to assess the cytotoxic effect of CDDP on TCam‐2 cells with different autophagy and TDRG1 expression levels. According to our previous study, we have determined the IC25 (2.55 μmol/L) and IC50 (14.73 μmol/L) concentrations of cisplatin for TCam‐2 cell lines.^32^ Total number of 10^4^ cells were seeded to each well in a 96‐well plate and randomly assigned into six groups: TCam‐2 control, TCam‐2 + CDDP(IC25), TCam‐2 + CDDP(IC25) + TDRG‐1 overexpression, TCam‐2 + CDDP(IC25) + TDRG‐1 knockdown, TCam‐2 + CDDP(IC25) + 3‐MA (1 mmol/L; 3‐methyladenine is an autophagy inhibitor) and TCam‐2 + CDDP(IC50). After different time of incubation (0, 12, 24, 48, 72, 96 hours), the cells then were incubated with 20 mL MTT solution with the final concentration of 5 mg/mL (Sigma‐Aldrich) for another 2 hours in the incubator. DMSO was then added to each well and stirred gently to dissolve the purple‐blue formazan crystals. Finally, the absorbance of each well at 570 nm (A570) was measured by Microplate Reader (Bio‐tek elx‐800).

### 2.8 Cell apoptosis analysis

The cell apoptosis was measured by Annexin V‐FITC (fluorescein isothiocyanate)/propidium iodide (PI) staining.^31^ After 72 hours of different treatment, cells were collected. After washed with cold phosphate‐buffered saline (PBS), cells then labelled with Annexin V and PI with Annexin V‐FITC apoptosis detection Kit (Beyotime Biotechnology) in the dark. Then FACSCalibur flow cytometry (BD Biosciences) was performed to analyse cell apoptosis.

### 2.9 Western blotting

For Western blotting (WB), the mouse xenograft tumours and TCam‐2 cells were lysed in lysis buffer containing protease inhibitors for 30 minutes. After centrifugation, the supernatants were isolated and protein concentration was determined using bicinchoninic acid assay (Beyotime Biotechnology). An equivalent amount of protein (20 μg) of each sample was separated by 10% SDS‐PAGE and then transferred to PVDF membranes. Then blocked the membranes with 5% non‐fat dry milk containing 0.1% Tween‐20 in Tris‐buffered saline (TBS‐T) at room temperature for 1 hour and then incubated with primary antibodies (Table 1) at 4°C overnight. After incubated in secondary antibody, immunoreactivity was detected by the enhanced chemiluminescence method (Thermo Scientific).

**Table 1:** Information of antibody used in Western blot

Primary antibodies	MW (kD)	Dilution	Company	Secondary antibodies	Dilution
LC3‐I/II	16/14	1:400	Abcam	Goat anti‐rabbit IgG/HRP	1:4000
TDRG‐1	11	1:1000	Acris	Goat anti‐rabbit IgG/HRP	1:4000
PI3K/p110β	110	1:1000	CST	Goat anti‐rabbit IgG/HRP	1:4000
PI3K III/VPS34	100	1:1000	CST	Goat anti‐rabbit IgG/HRP	1:4000
Beclin‐1	52	1:2000	ABCAM	Goat anti‐rabbit IgG/HRP	1:4000
ATG14L	55	1:1000	Abcam	Goat anti‐rabbit IgG/HRP	1:4000
active Caspase‐3	17	1:1000	Abcam	Goat anti‐rabbit IgG/HRP	1:4000
RAB5A	24	1:1000	ptgcn	Goat anti‐rabbit IgG/HRP	1:4000
GAPDH	37	1:800	SANTA	Goat anti‐mouse IgG/HRP	1:8000

Abbreviation: MV, molecular weight.

### 2.10 Immunofluorescence staining

For immunofluorescence staining, after 72 hours of different treatment, cells were fixed in cold methanol for 5 minutes and permeabilized with 0.1% Triton X‐100 (Sigma‐Aldrich) for 8 minutes at room temperature. Then, cells were blocked with 3% BSA in PBS for 1 hour and incubated with primary antibody against LC3‐II followed with secondary antibody (anti‐rabbit IgG labelled with Alexa 555). Then stained the nucleus with DAPI. Finally, cells were observed by confocal microscope.

### 2.11 Xenografts

Twenty‐four 4‐5 weeks old male athymic BALB/c nude mice were purchased from the Institute of Experimental Animals of Central South University. All mice were free to get standard laboratory mouse food and water. After one week be kept in a sterile environment, TCam‐2 cells (1 × 10^6^ suspended in 200 μL medium) with siRNA control, TDRG1‐overexpressing or TDRG1 knockdown were randomly injected into groin area subcutaneously. After one week, mice were divided into four groups (six mice per group) based on the level of TDRG1 expression. Meanwhile, 3‐MA (3 mg/kg, i.p., once per week for 4 weeks) and CDDP (3 mg/kg, i.p., once per week for 4 weeks) were managed as followed: CDDP Control group, CDDP + TDRG1 overexpression group, CDDP + TDRG1 knockdown group, CDDP + 3‐MA group. The mice were followed up to 31 days then sacrificed, then the tumours were removed. The tumour volume was calculated according to the formula length × width^2^ × 0.5.

### 2.12 Immunohistochemistry analysis

Tumour sections of xenografts were deparaffinized in xylene, then rehydrated in ethanol and finally rehydrated in double‐distilled water. The sections were then placed in 0.01 mol/L citrate buffer (pH 6.0), and microwave heated for 20 minutes to retrieval antigen. The sections were blocked with 3% goat serum for 60 minutes and then incubated with anti‐Ki67 at 4°C overnight. The primary antibody was observed by light microscopy with SP method following the instructions (Maixin Biotechnology). According to the intensity and proportion of immunoreactive cells, protein expression levels were classified semi‐quantitatively.

### 2.13 Statistical analysis

All experiments were repeated in triplicate and all data were presented as the average of three independent experiments. Data were analysed with Prism 5 (GraphPad Software) and presented as mean ± standard deviation (SD). Student's *t* test was used to analyse the statistical significance of the difference between the two groups. One‐way ANOVA was performed for statistical significance among multiple groups.

## 3 RESULTS

### 3.1 Establishment of seminoma TCam‐2 cells with TDRG1, PI3K/p110β or Rab5 knockdown and TDRG1 overexpression

In our previous study, we established TDRG1 knockdown and overexpressing seminoma TCam‐2 cell lines.^32^ In the present study, we generated PI3K/p110β or Rab5 siRNA stably transfected TCam‐2 cells. Successful transfection was confirmed by GFP expression using fluorescence microscopy (Figure S1). Moreover, Western blot demonstrated that siRNA against PI3K/p110β or Rab5 was efficient and specific in knocking down the respective gene at the protein level (Figure S1). Thus, cell models for PI3K/p110β and Rab5 knockdown were set up successfully.

### 3.2 TDRG1 promotes autophagy in testicular seminoma and TCam‐2 cells

**Figure 1:**
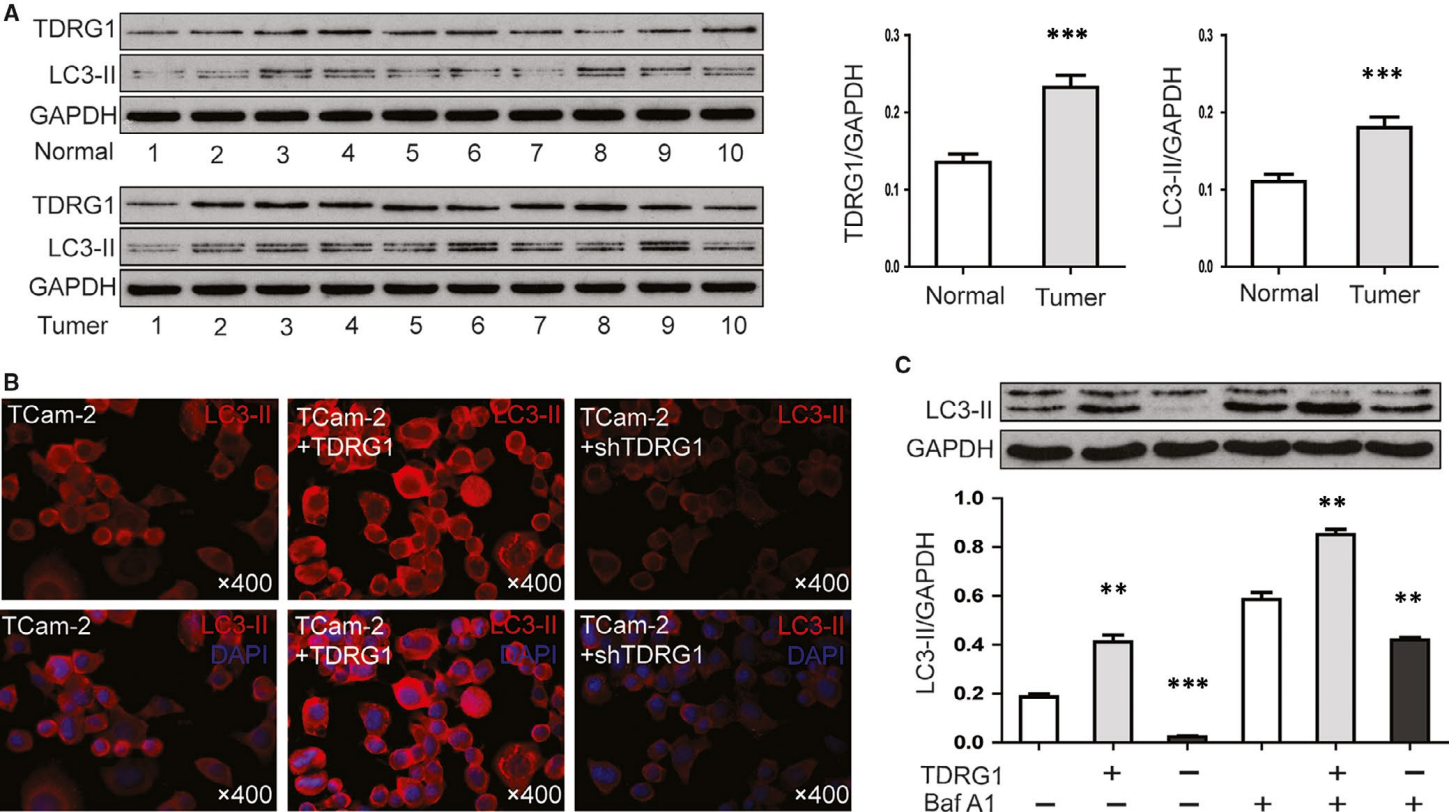
Testis developmental related gene 1 (TDRG1) promotes autophagy in both seminoma tissues and TCam‐2 cells. A, Western blot were performed to detected the expression of TDRG1 and LC3‐II in testicular seminoma tissues (n = 10) and normal testicular tissues (n = 10). B, Immunofluorescence imaging of LC3‐II(red) in TCam‐2 cells and TCam‐2 cells with TDRG1 overexpression or knockdown. C, TCam‐2 cells and TCam‐2 cells with TDRG1 overexpression or knockdown were left untreated or treated with 200 nmol/L bafilomycin A1 (Baf A1) for 6 h. Relative levels of LC3‐II against GAPDH from three independent experiments are shown. (Error bars represent SD, **P < .01, ***P < .001)

Expression of TDRG1 and LC3‐II (microtubule‐associated protein 1 light chain 3B) at the protein level of testicular seminoma tissues and normal testicular tissues was detected by Western blot. In normal testicular tissue, the TDRG1/GAPDH and LC3‐II/GAPDH ratios were 0.14 ± 0.01 and 0.11 ± 0.01, respectively. In testicular seminoma tissues, the TDRG1/GAPDH ratio increased to 0.23 ± 0.02 (*P* < .001) and the LC3‐II/GAPDH ratio increased to 0.18 ± 0.01 (*P *< .001; Figure 1A).

To examined whether TDRG1 promotes autophagy of TCam‐2 cells, expression level of LC3‐II in TCam‐2 cells and TCam‐2 cells with TDRG1 knocked down or overexpressed was compared. By immunofluorescence imaging, the fluorescence intensity of LC3‐II was increased in TDRG1‐overexpressing TCam‐2 cells and decreased in TDRG1 knockdown (Figure 1B). We also applied the lysosomal inhibitor Baf A1 to obtain a more accurate measure of the level of autophagy. First, we examined the response of TCam‐2 cells to a range of Baf A1 concentrations (Figure S2) and found 200 nmol/L to be the optimal concentration for lysosomal inhibition. Western blot analysis further confirmed that overexpression of TDRG1 promotes LC3‐II expression and knockdown of TDRG1 inhibits LC3‐II expression in TCam‐2 cells, with or without Baf A1 treatment (Figure 1C). The LC3‐II/GAPDH ratio was 0.18 ± 0.01 in TCam‐2 control cells, increased to 0.41 ± 0.03 in TDRG1‐overexpressing cells (*P* < .01) and decreased to 0.02 ± 0.003 in TDRG1 knockdown cells (*P* < .001). After adding Baf A1, the LC3‐II/GAPDH ratio was 0.59 ± 0.03 in TCam‐2 control cells, increased to 0.85 ± 0.03 in TDRG1‐overexpressing cells (*P *< .01) and decreased to 0.42 ± 0.01 in TDRG1 knockdown cells (*P *< .01).

### 3.3 TDRG1 promotes autophagy through activation of p110β/Rab5/Vps34 (PI3‐Kinase Class III)

**Figure 2:**
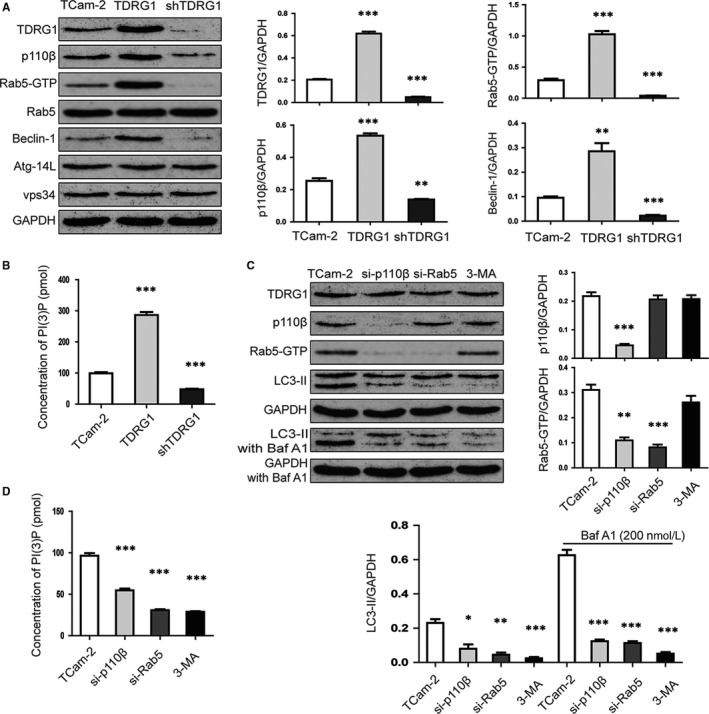
Testis developmental related gene 1 (TDRG1) promotes autophagy through p110β/Rab5/Vps34 (PI3‐Kinase Class III) activation. A, Protein expression of TDRG1, p110β, Rab5‐GTP, Rab5, Beclin‐1, Atg‐14L and Vps34 in TCam‐2 cells was measured by Western blotting at 72 h after transfection with constructs to overexpress of knockdown TDRG1. GAPDH served as the internal control. B, Vps34 activity was measured by analysing PI(3)P production by the ELISA described in Section 2. C, Protein expression of TDRG1, p110β, Rab5‐GTP and LC3‐II (untreated or treated with 200 nmol/L Baf A1 for 6 h) was measured by protein gel blotting 72 h after transfection with siRNA to block the expression of p110β and Rab5 or treatment with 3‐MA (autophagy inhibitor). GAPDH served as the internal control. D, Vps34 activity was measured by analysing PI(3)P production by the ELISA described in Section 2. (Data from three independent experiments, error bars represent SD, *P < .05, **P < .01, ***P < .001)

To gain insight into the mechanism underlying the autophagy triggered by TDRG1, we focused on the p110β/Rab5/Vps34 (PI3‐Kinase Class III) pathway and the expression levels of relevant proteins, including Beclin‐1, a regulator of autophagy. As shown in Figure 2A, in TCam‐2 control cells, the TDRG1/GAPDH ratio was 0.21 ± 0.01, the p110β/GAPDH ratio was 0.25 ± 0.02, the Beclin‐1/GAPDH ratio was 0.09 ± 0.01, and the Rab5‐GTP/GAPDH ratio was 0.29 ± 0.02. In the TDRG1‐overexpressing cells, the TDRG1/GAPDH ratio increased to 0.62 ± 0.02 (*P* < .001), the p110β/GAPDH ratio increased to 0.53 ± 0.01 (*P* < .001), the Beclin‐1/GAPDH ratio increased to 0.29 ± 0.03 (*P *< .01), and the Rab5‐GTP/ GAPDH ratio increased to 1.03 ± 0.05 (*P *< .001). In the TDRG1 knockdown cells, the TDRG1/GAPDH ratio decreased to 0.05 ± 0.005 (*P* < .01), the p110β/GAPDH ratio decreased to 0.13 ± 0.003 (*P *< .01), the Beclin‐1/GAPDH ratio decreased to 0.02 ± 0.004 (*P *< .001), and the Rab5‐GTP/GAPDH ratio decreased to 0.04 ± 0.01 (*P *< .001). There was no significant change in the expression of total Rab5, Atg‐14L and Vps34. At the same time, we checked the activity of Vps34 by using a Class III PI3K ELISA Kit (Echelon, Lot# K 3000). We found that the concentration of PI(3)P, which reflects the activity of Vps34, was 99.26 ± 6.29 pmol in TCam‐2 control cells, increased to 286.35 ± 17.16 pmol (*P *< .001) in TDRG1‐overexpressing cells and decreased to 47.63 ± 3.12 pmol (*P *< .001) in TDRG1 knockdown cells (Figure 2B). The results of Western blotting suggest that TDRG1 can promote the expression of p110β, which in turn can convert Rab5 to its active form (Rab5‐GTP) to further activate Vps34 and promote autophagy.

We tested this hypothesis by using TCam‐2 cells with p110β or Rab5 knockdown. As showed in Figure 2C,D, in TCam‐2 control cells, the Rab5‐GTP/GAPDH ratio was 0.31 ± 0.02, the concentration of PI(3)P was 96.43 ± 5.42 pmol, and the LC3‐II/GAPDH ratio was 0.23 ± 0.02 without Baf A1 and 0.63 ± 0.03 with Baf A1. In the TCam‐2 cells with 110β knockdown, the expression of TDRG1 did not differ from that in TCam‐2 control cells, but the expression of p110β was decreased, as expected. As a result, the Rab5‐GTP/GAPDH ratio decreased to 0.11 ± 0.01 (*P *< .01), the concentration of PI(3)P decreased to 54.67 ± 3.78 pmol (*P *< .001), and the LC3‐II/GAPDH ratio decreased to 0.08 ± 0.03 (*P* < .05) without Baf A1 and to 0.12 ± 0.01 (*P *< .001) with Baf A1. These results indicate that TDRG1 cannot activate Rab5 without p110β, which means TDRG1 activates Rab5 through p110β. In TCam‐2 cells with Rab5 knockdown, the expression of TDRG1 and p110β showed no difference from TCam‐2 control cells, but the expression of Rab5‐GTP was decreased. As a result, the concentration of PI(3)P decreased to 30.84 ± 2.01 pmol (*P* < .001), and the LC3‐II/GAPDH ratio decreased to 0.04 ± 0.01 (*P *< .01) without Baf A1 and to 0.11 ± 0.02 (*P *< .001) with Baf A1. These results indicate that TDRG1 cannot activate Vps34 without Rab5, which means that TDRG1 activates Vps34 through Rab5. When we used 3‐MA, an inhibitor of Vps34, the concentration of PI(3)P decreased to 28.76 ± 1.36 pmol (*P *< .001) and the LC3‐II/GAPDH ratio decreased to 0.02 ± 0.01 (*P *< .001) without Baf A1 and to 0.05 ± 0.01 (*P *< .001) with Baf A1. These results confirm that TDRG1 promotes autophagy through p110β/Rab5/Vps34 (PI3‐Kinase Class III) activation.

### 3.4 TDRG1 and autophagy regulate cell viability and apoptosis in response to CDDP treatment in TCam‐2 cells

**Figure 3:**
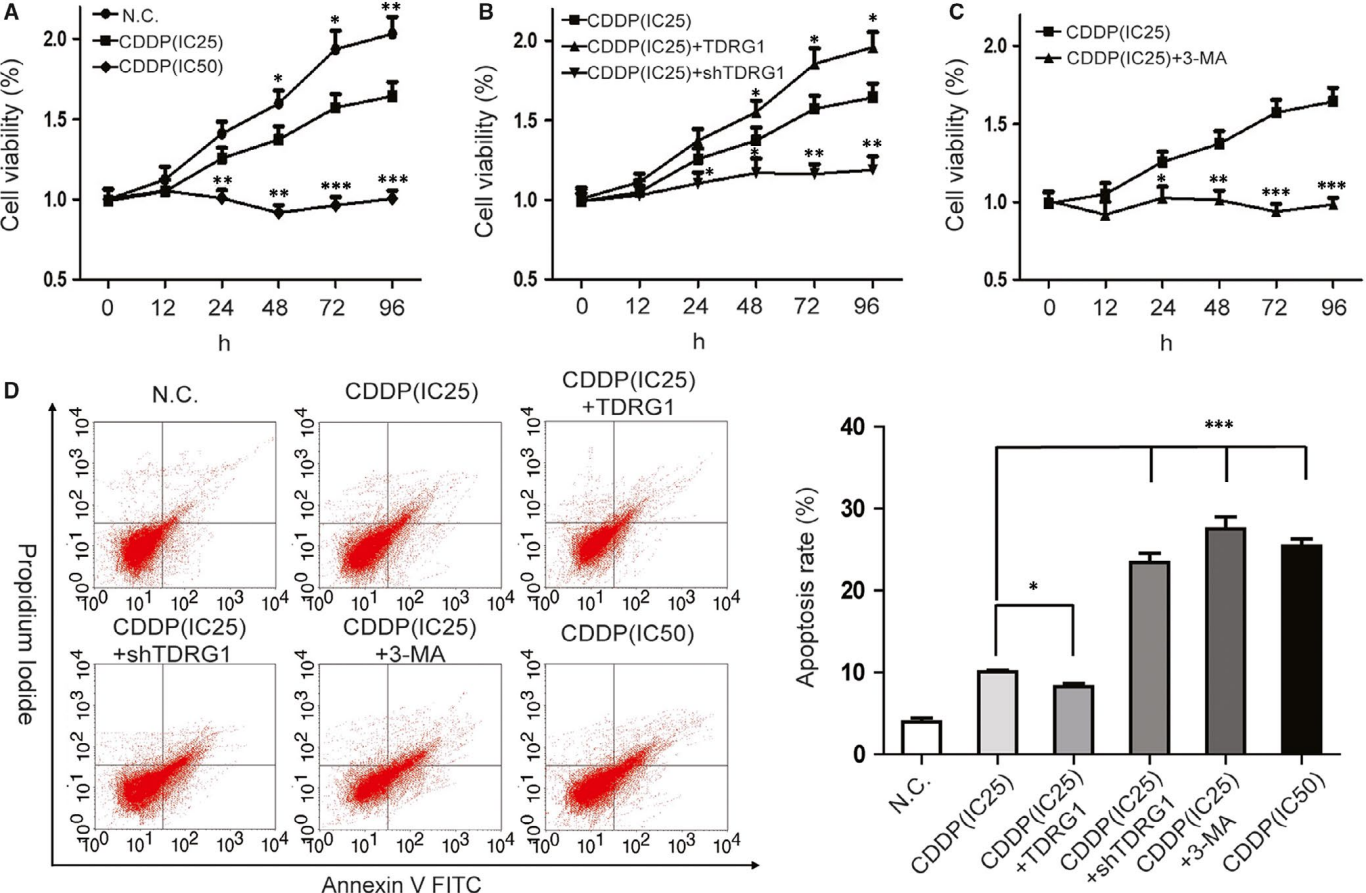
Testis developmental related gene 1 (TDRG1) and autophagy regulate cell viability and apoptosis in response to *cis*‐dichlorodiammine platinum (CDDP) treatment in TCam‐2 cells. A, MTT assays were used to confirm the effects of different concentrations of CDDP on the viability of TCam‐2 cells. B, MTT assays were performed to confirm the effects of different expression levels of TDRG1 on the viability of TCam‐2 cells following CDDP(IC25) treatment. C, MTT assays were used to confirm the effects of autophagy inhibition with 3‐MA on the viability of TCam‐2 cells following CDDP(IC25) treatment. D, Left: Cells were treated with different concentrations of CDDP or subjected to up‐ or down‐regulation of TDRG1 or treated with the autophagy inhibitor 3‐MA for 72 h, followed by staining with Annexin V‐fluorescein isothiocyanate and propidium iodide and analysed by flow cytometry. Right: Quantitative summary of the early apoptosis rate in the different treatment groups. Compared as indicated. (Data from three independent experiments, error bars represent SD, *P < .05, **P < .01, ***P < .001)

Based on our previous study,^32^ we chose a CDDP concentration of IC25 to treat TCam‐2 cells to investigate their chemosensitivity to CDDP, and IC50 as a positive control. MTT assays were employed to quantify the chemosensitivity of TCam‐2 cells to CDDP. After incubation with the two concentrations (IC25 and IC50) of CDDP for 48 hours, the cell viability of TCam‐2 cells was markedly reduced in the IC25 dose compared with the negative control (NC) and further reduced in the IC50 dose (Figure 3A). Furthermore, knockdown of TDRG1 led to a marked reduction in the viability of cells treated with CDDP(IC25), bringing the survival close to that of TCam‐2 cells treated with CDDP(IC50). In contrast, overexpression of TDRG1 significantly increased the cell viability, bringing it close to that of the NC group (Figure 3B). We also examined the effect of inhibition of autophagy on the chemosensitivity of TCam‐2 cells to CDDP(IC25). We found that inhibition of autophagy by applying 3‐MA significantly reduced the viability of TCam‐2 cells treated with CDDP(IC25), lowering it to the level induced by CDDP(IC50) (Figure 3C).

Since control of apoptosis may conducive to the regulatory effect of TDRG1 on CDDP chemosensitivity, flow cytometric analysis was performed to investigate this possibility (Figure 3D). The apoptosis proportion was very low (3.96 ± 0.47%) in the NC group, increased to 10.09 ± 0.95% (*P* < .001) in the CDDP(IC25) group and to 25.44 ± 1.47% (*P *< .001) in the CDDP(IC50) group. It means that CDDP can increase the level of apoptosis in TCam‐2 in a concentration‐depend manner. Furthermore, after incubation with CDDP(IC25), the level of apoptosis decreased to 8.26 ± 0.40% (*P* < .05) in TDRG1‐overexpressing cells and increased to 23.43 ± 1.11% (*P* < .001) in TDRG1 knockdown cells (Figure 3D). Similarly, in the 3‐MA group, the apoptosis level increased to 27.52 ± 1.46% (*P *< .001).

Based on these data, we assume that in the case of CDDP(IC25) treatment, TDRG1 overexpression attenuates the chemosensitivity of TCam‐2 cells to CDDP, and TDRG1 knockdown or autophagy inhibition enhances the chemosensitivity. The promoting effect of TDRG1 knockdown and autophagy inhibition is similar; and, finally, the effect on apoptosis and proliferation is close to that exerted by CDDP(IC50).

### 3.5 TDRG1 regulates the chemosensitivity of TCam‐2 cells to CDDP via autophagy

**Figure 4:**
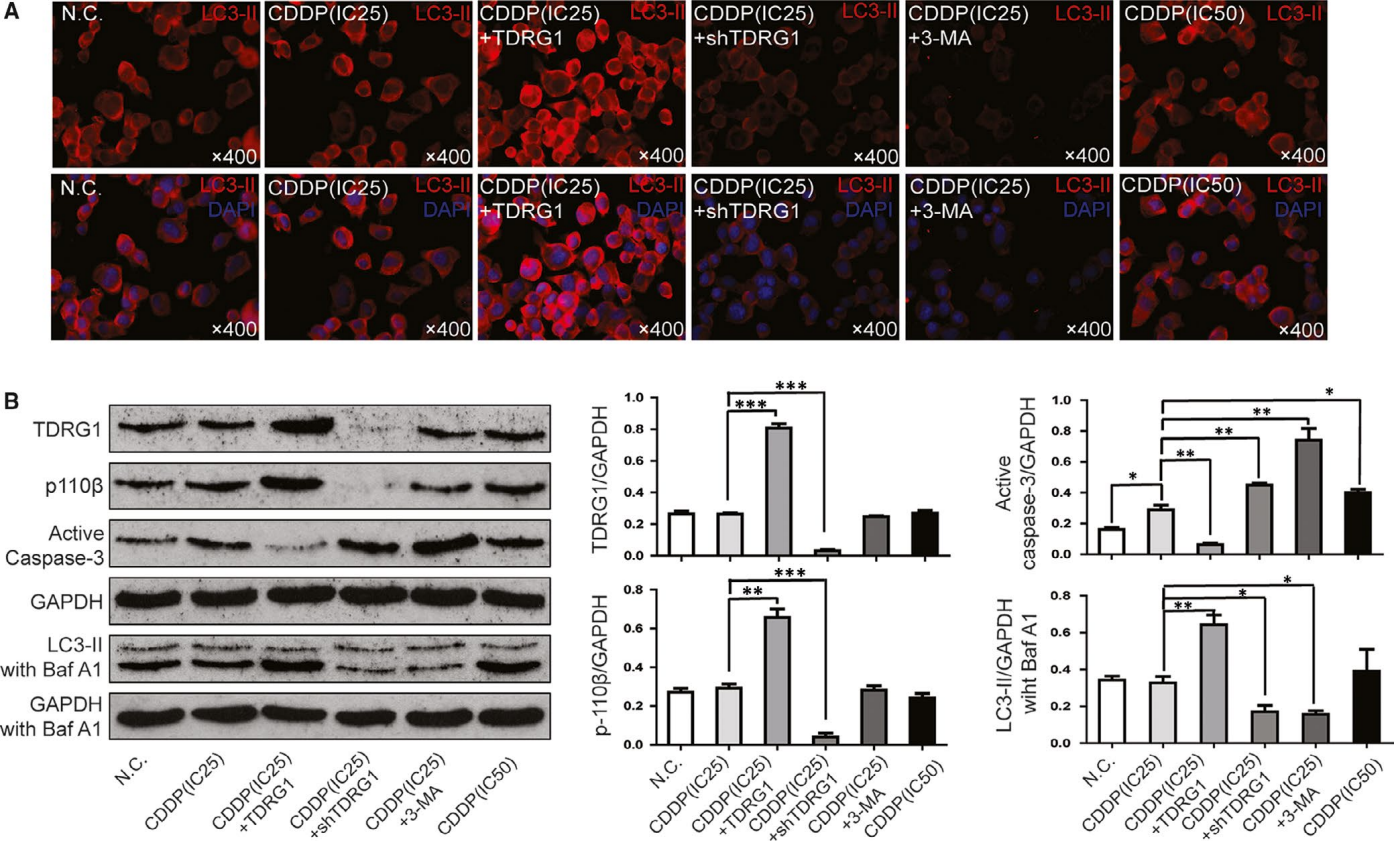
Testis developmental related gene 1 (TDRG1) regulates chemosensitivity of TCam‐2 cells to *cis*‐dichlorodiammine platinum (CDDP) through autophagy. A, Immunofluorescence images of LC3‐II (red) in TCam‐2 cells treated with different concentrations of CDDP or subjected to up‐ or down‐regulation of TDRG1 or treated with the autophagy inhibitor 3‐MA for 72 h. B, Protein expression of TDRG1, p110β, LC3‐II and active caspase 3 in TCam‐2 cells with or without Baf A1 treatment was measured by Western blotting at 72 h after treatment with different concentrations of CDDP or with different expression levels of TDRG1 or autophagy inhibition with 3‐MA. GAPDH served as an internal control. (Data from three independent experiments, error bars represent SD, *P < .05, **P < .01, ***P < .001)

As TDRG1 can regulate autophagy, and because autophagy activation may be an important factor in promoting resistance to chemotherapy,^18^, ^19^ we next sought to determine whether autophagy is involved. To do this, we examined the expression levels of autophagic biomarkers in TCam‐2 cells in which TDRG1 was either knocked down or overexpressed. By IF microscopy, the fluorescence intensity of LC3‐II was increased in TCam‐2 cells overexpressing TDRG1, and it was decreased in TDRG1 knockdown cells and in cells treated with 3‐MA and CDDP(IC25); in contrast, there were no significant differences between the NC, CDDP(IC25) and CDDP(IC50) groups (Figure 4A). Western blot measurements further confirmed these findings (Figure 4B). After incubated in Baf A1(200 nmol/L), the LC3‐II/GAPDH ratio was 0.33 ± 0.04 in TCam‐2 cells, increased to 0.64 ± 0.05 in TDRG1‐overexpressing cells (*P *< .01) and decreased to 0.17 ± 0.03 in TDRG1 knockdown cells (*P *< .05) and to 0.16 ± 0.02 in 3‐MA‐treated cells (*P *< .05). There was no significant difference in the ratios between the TDRG1 knockdown and the 3‐MA‐treated cells, indicating that the effect of TDRG1 knockdown on autophagy inhibition is as strong as that caused by 3‐MA.

We next examined the expression of p110β to determine if TDRG1 regulates autophagy through p110β/Rab5/Vps34 (PI3‐Kinase Class III) when treated with CDDP(IC25). The expression levels of p110β among the N.C, CDDP(IC25), 3‐MA and CDDP(IC50) groups were not significantly different. The p110β/GAPDH ratio was 0.29 ± 0.02 in TCam‐2 cells, increased to 0.66 ± 0.04 in TDRG1‐overexpressing cells (*P *< .01) and decreased to 0.04 ± 0.01 in TDRG1 knockdown cells (*P *< .001; Figure 4B). The level of p110β expression in TCam‐2 cells treated with CDDP(IC25) was similar to that in cells without CDDP treatment, which indicates that TDRG1 regulates autophagy through p110β/Rab5/Vps34 (PI3‐Kinase Class III) independent of CDDP treatment.

As caspase‐3 is a key effector in apoptosis, we next examined the expression of activated caspase‐3. As shown in Figure 4B, the ratio of activated caspase‐3 to GAPDH was very low (around 0.16 ± 0.01) in the NC group, increased to 0.28 ± 0.03 (*P *< .05) in CDDP(IC25) group and to 0.39 ± 0.03 (*P *< .01) in the CDDP(IC50) group (Figure 4B). This result indicates that CDDP can increase the level of apoptosis of TCam‐2 cells in a concentration‐dependent manner. After incubation in CDDP(IC25), the ratio of activated caspase‐3 to GAPDH decreased to 0.06 ± 0.01(*P *< .01) in the TDRG1‐overexpressing group and increased to 0.45 ± 0.01 (*P *< .01) in the TDRG1 knockdown group (Figure 4B). A similar result was observed in the 3‐MA‐treated group, where the ratio increased to 0.74 ± 0.08 (*P *< .001). As caspase‐3 is very important in the autophagy‐associated apoptosis pathway, these results indicate that TDRG1 regulates apoptosis through its effects on autophagy.

### 3.6 TDRG1 regulates seminoma growth via autophagy in response to CDDP in vivo

**Figure 5:**
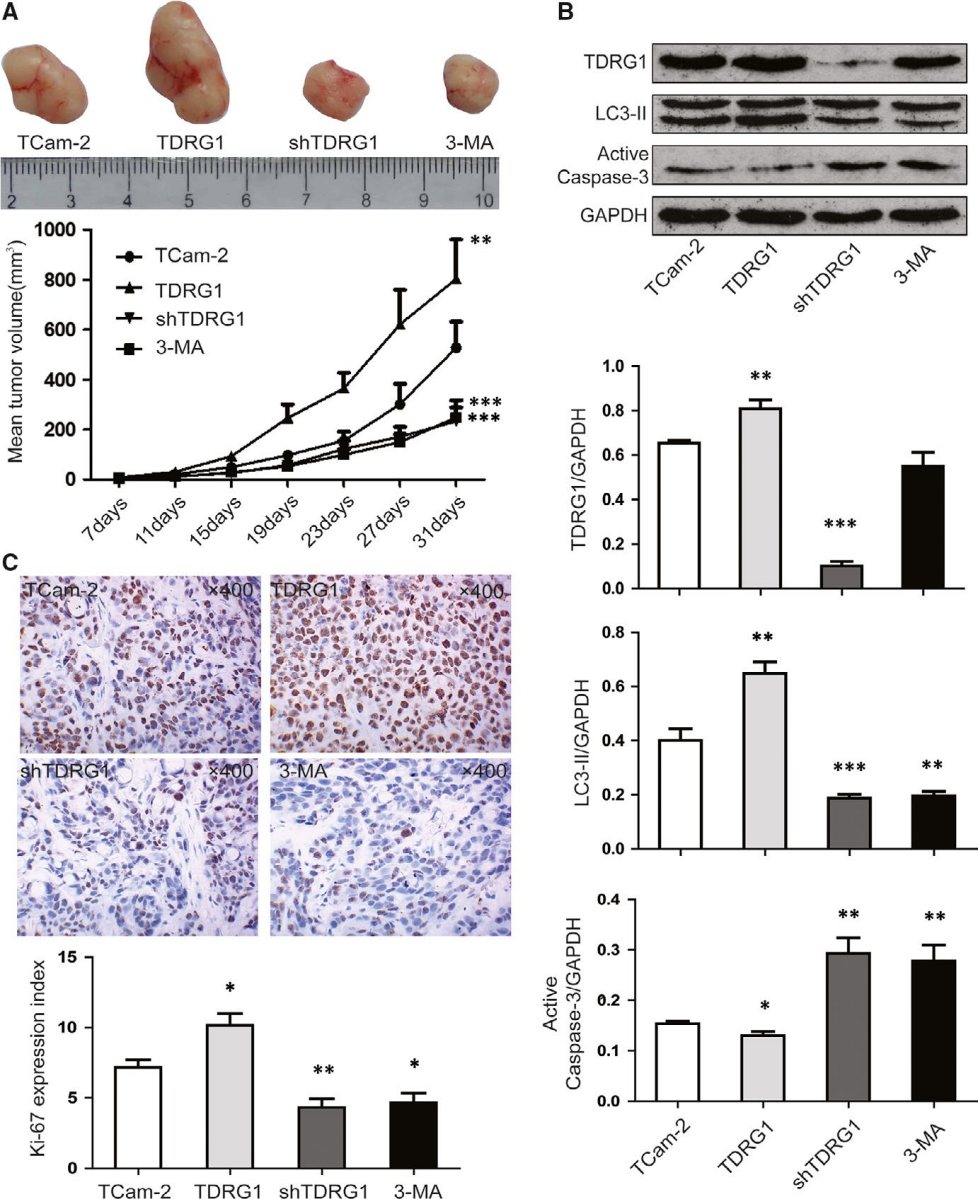
Testis developmental related gene 1 regulates seminoma growth through autophagy in response to *cis*‐dichlorodiammine platinum treatment in vivo. A, Images of representative tumours in nude mice from different treatment groups and growth curves of transplanted tumours in nude mice (n = 6). B, Protein lysates were collected from mouse xenograft tumours from different treatment groups, and the levels of TDRG1, LC3‐II and active caspase‐3 were measured by Western blotting. GAPDH served as a loading control. C, Representative images of immunohistochemistry analysis of Ki‐67, magnification 200×. The expression index of Ki‐67 in the different groups was analysed (error bars represent SD, *P < .05, **P < .01, ***P < .001)

Xenograft tumour model in male BALB/c nude mice was established to investigate the effect of TDRG1 on the chemosensitivity of seminoma cells to CDDP through autophagy in vivo. As shown in Figure 5A, after four cycles of CDDP(IC25) treatment, the mean volume of tumours with TDRG1 overexpression was significantly bigger and TDRG1 knockdown or 3‐MA treatment obviously smaller than control.

To check the molecular mechanism, we performed Western blot analyses to check the autophagy and a related apoptotic pathway. As shown in Figure 5B, the LC3‐II/GAPDH ratio was 0.40 ± 0.04 in the CDDP group, increased to 0.65 ± 0.04 in TDRG1‐overexpressing cells (*P *< .01) and decreased to 0.19 ± 0.01 in TDRG1 knockdown cells (*P *< .001) and to 0.20 ± 0.02 in 3‐MA‐treated cells (*P *< .01). The activated caspase‐3/GAPDH ratio was 0.15 ± 0.01 in the TCam‐2 group, decreased to 0.06 ± 0.01 (*P *< .05) in the TDRG1 overexpression group and increased to 0.29 ± 0.03 (*P *< .01) in the TDRG1 knockdown group and to 0.28 ± 0.03 in the 3‐MA group (*P *< .01; Figure 5B). Furthermore, expression of Ki‐67(a cell proliferation marker^33^) in the TDRG1 overexpression group was increased significantly compared to the CDDP group, while the TDRG1 knockdown and 3‐MA CDDP groups were markedly decreased (Figure 5C).

## 4 DISCUSSION

Chemotherapy is widely used for treatment to many kinds of cancers, including seminoma. Indeed, CDDP is one of the most commonly used anti‐cancer agents in seminoma patients due to the high sensitivity.^34^, ^35^ Most seminoma patients, even those in later clinical stages, could have a good outcome with chemotherapy based on CDDP. However, patients who are not sensitive to CDDP experience pain and inevitably succumb to the disease.^7^, ^8^, ^36^ Although the mechanisms that confer resistance or sensitivity of seminoma cells to CDDP are unclear, several have been postulated.^37^, ^38^ The TDRG1 gene was involved in one of the mechanisms. In our model, we hypothesized that H19 long non‐coding RNA promotes the expression of TDRG1 by sequestering miRNA‐106b‐5p.^39^ In turn, up‐regulated TDRG1 promotes resistance to CDDP through PI3K/Akt/mTOR signalling and the mitochondria‐mediated apoptotic pathway.^32^ In the present study, we aimed to investigate whether TDRG1 protein regulates autophagy and chemosensitivity to CDDP in seminoma cells.

Autophagy is an important cellular process for maintaining proper cell function and homeostasis in response to various stress conditions. We examined the expression of TDRG1 and the level of autophagy in both normal testicular tissues and seminoma. The data indicated that the expression levels of LC‐3II, an autophagy marker, and TDRG1 were higher in seminoma than in normal tissue, demonstrate a potential relationship between TDRG1 and autophagy in seminoma. Furthermore, we confirmed that TDRG1 overexpression up‐regulates and TDRG1 knockdown down‐regulates the level of autophagy in TCam‐2 cells. These results indicate that TDRG1 upregulation in seminoma is likely a key driver of the high level of autophagy in this kind of tumour.

To further investigate the mechanism by which TDRG1 regulates autophagy, we checked the p110β/Rab5/Vps34 (PI3‐Kinase Class III) pathway. The small GTPase Rab5, which in its GTP‐bound form is critical for endocytic trafficking,^40^, ^41^ also has important role for autophagosome formation through interacting with the Vps34‐Beclin 1 complex.^42^ Rab5 promotes autophagy by recruiting Vps34 and facilitating Vps34’s localized activity to early endosomes. Recently, two other studies reported that p110β promotes autophagy by binding to Rab5, a kinase‐independent manner.^29^, ^30^ The p110β‐Rab5 interaction protects Rab5‐GTP and increases the amount of activated Rab5, then promotes Rab5 interactions with Vps34, which finally induces autophagy.^29^, ^30^ In this study, we found that the expression of p110β and Rab5, the activity of Vps34, and the autophagy flux were all increased in TCam‐2 cells overexpressing TDRG1 and were decreased in TDRG1 knockdown cells. More importantly, knockdown of p110β or Rab5 in TCam‐2 cells, resulted in significant reductions in the activity of Vps34 and autophagy flux, while the TDRG1 level stayed the same. These data support a model in which TDRG1 increases the expression of p110β, which in turn binds to and stabilizes Rab5, which then activates Vps34 to promote autophagy in TCam‐2 cells.

It is believed that autophagy has an opposing role in cancer. In the field of chemotherapy, many studies have proved that autophagy confers protection to tumour cells from apoptosis and the toxicity of chemotherapy agents; thus, to some extent, inhibition of apoptosis by autophagy may result in chemoresistance.^20^, ^21^, ^22^, ^23^, ^43^, ^44^, ^45^, ^46^ On the other hand, some other studies have shown that autophagy can trigger an autophagic death pathway, which is the main mechanism of cell death in response to certain chemotherapeutics.^17^, ^47^, ^48^, ^49^ Therefore, autophagy is considered to be an important target of cancer therapy,^50^ and autophagy‐modulating agents such as amodiaquine, hydroxychloroquine and chloroquine have been widely used for tumour therapy.^51^, ^52^, ^53^, ^54^ Here, we demonstrated that autophagy has a protective effect on seminoma cells and may lead to CDDP resistance both in vitro and in vivo. In the in vitro experiments, inhibition of autophagy by 3‐MA can induced apoptosis and reduced the viability of TCam‐2 cells treated with CDDP. Our in vivo experiments verified these findings, since inhibition of autophagy by 3‐MA down‐regulated the growth of xenograft seminoma cancer, decreased the expression level of the cell proliferation marker Ki‐67, and increased the expression of activated capsase3 involved in apoptosis.

**Figure 6:**
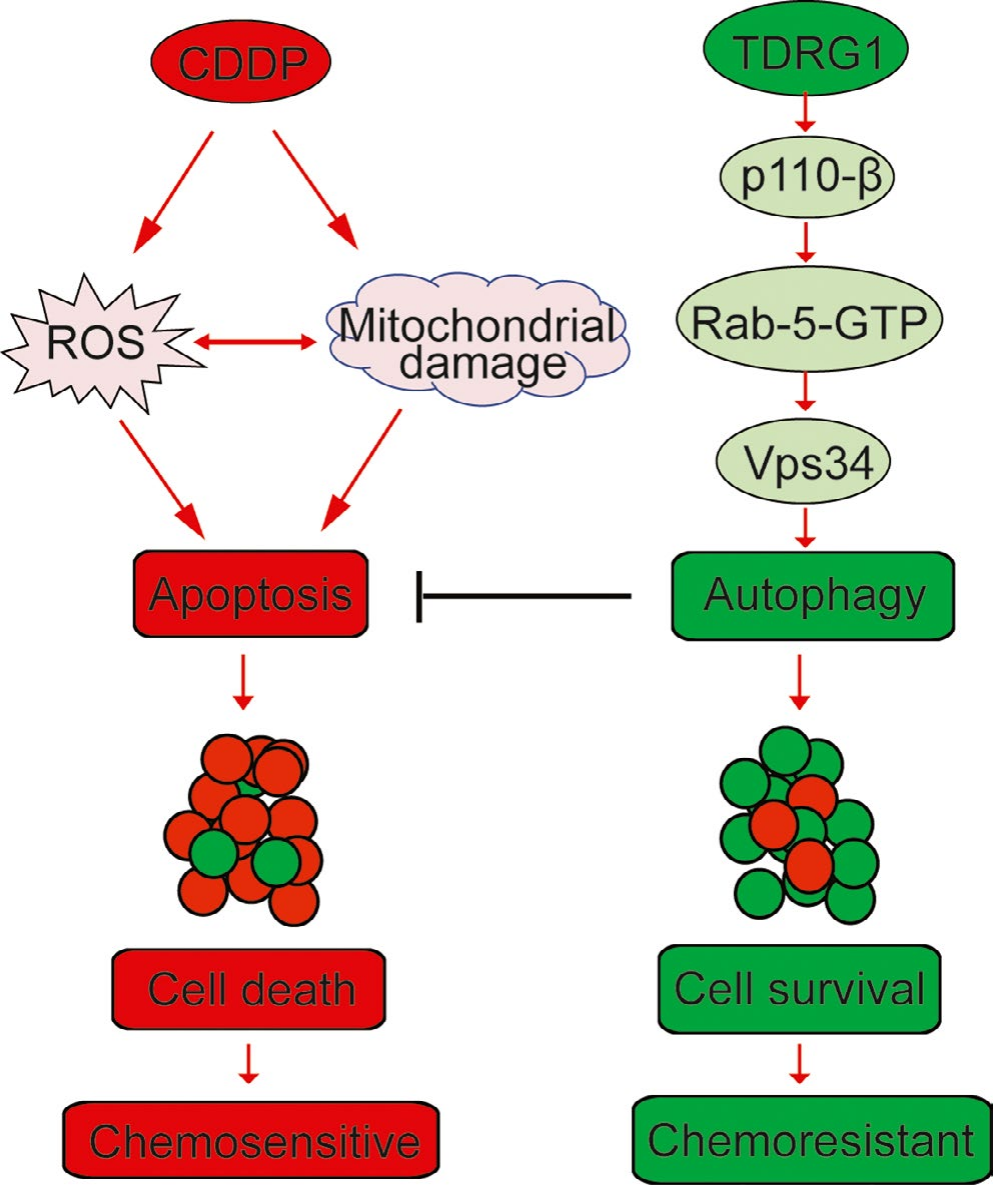
Schematic representation of the role of testis developmental related gene 1 in regulating autophagy of TCam‐2 cells during *cis*‐dichlorodiammine platinum treatment

We next investigated the mechanisms by which TDRG1 mediates CDDP chemosensitivity through autophagy. In the in vitro experiments, TCam‐2 cells overexpressing TDRG1 had a high level of p110β, sufficient to promote autophagy flux through the p110β/Rab5/Vps34 (PI3‐Kinase Class III) pathway during exposure to CDDP. We then confirmed that autophagy can promote cell viability (as measured by MTT assay) and inhibit apoptosis (as assayed by flow cytometric analysis and expression of activated capsase3). TCam‐2 cells with TDRG1 knockdown showed exactly the opposite results. Moreover, these findings were verified by our in vivo experiments, since TDRG1 overexpression increased the expression levels of Ki‐67 and activated capsase3 and stimulated the growth of xenograft seminoma cancer, whereas TDRG1 knockdown had the opposite effects. These results indicate that during treatment with CDDP, TDRG1 overexpression can promote autophagy, which in turn can inhibit apoptosis and promote cell viability, and lead to CDDP resistance both in vivo and in vitro. TDRG1 knockdown exerts the opposite effects, resulting in chemosensitivity to CDDP both in vivo and in vitro (Figure 6).

In summary, our findings show that high expression of TDRG1 supports a high level of autophagy in seminoma. TDRG1 regulates autophagy through the p110β/Rab5/Vps34 (PI3‐Kinase Class III) pathway in TCam‐2 cells. Autophagy has a protective effect on seminoma cells during chemotherapy and increased autophagy may lead to CDDP resistance. TDRG1 overexpression promotes autophagy and leads to CDDP resistance, whereas TDRG1 knockdown inhibits autophagy and ultimately promotes chemosensitivity to CDDP both in vivo and in vitro. This study has uncovered a novel role of TDRG1 in determining chemoresistance during CDDP treatment and provides potential therapeutic strategies for the treatment of human seminoma.

## CONFLICT OF INTEREST

The authors have no conflict of interest.

## AUTHORS' CONTRIBUTIONS

DP and YT designed the experiments. DP, JW, XJ, YX and YD performed the experiments and collected the data. DP, JW and YG analysis and interpretation of the data. RK provided the TCam‐2 cells. DP, JY and YT were the major contributors in writing the manuscript. All authors read and approved the manuscript.

## Data Availability

The data that support the findings of this study are available from the corresponding author upon reasonable request.
