# Supplementary material for: Testis developmental related gene 1 regulates the chemosensitivity of seminoma TCam‐2 cells to cisplatin via autophagy
Source: J Cell Mol Med. 2019 Sep 9;23(11):7773–84. doi: 10.1111/jcmm.14654 (PMC6815826; doi:10.1111/jcmm.14654)
Supplement: Supplementary file 1 [file JCMM-23-7773-s001.docx]

**Supplementary**

**
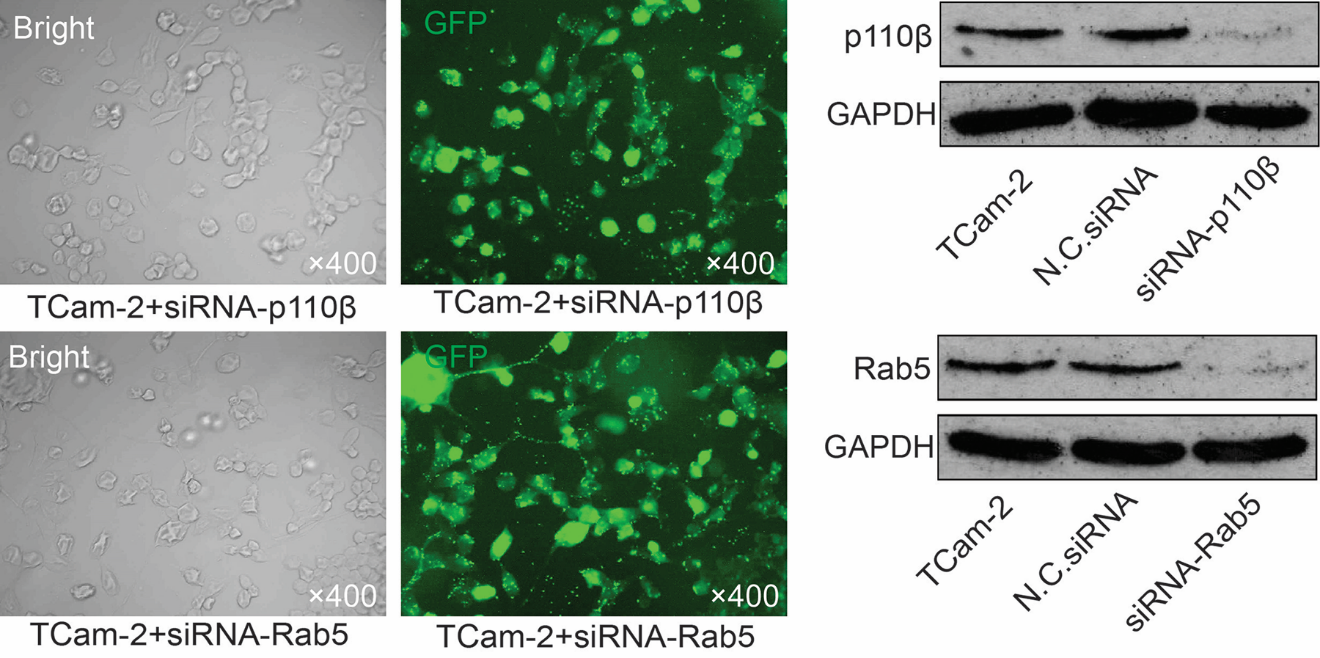
**

**Figure s1. Establishment of TCam-2 cells with** **p110β or Rab5 knockdown.** Successful transfection of p110β and Rab5 was confirmed by detecting GFP expression using laser confocal scanning microscopy (magnification, × 400). The protein expression levels of p110β and Rab5 in the transfected cells were detected by western blotting.

**
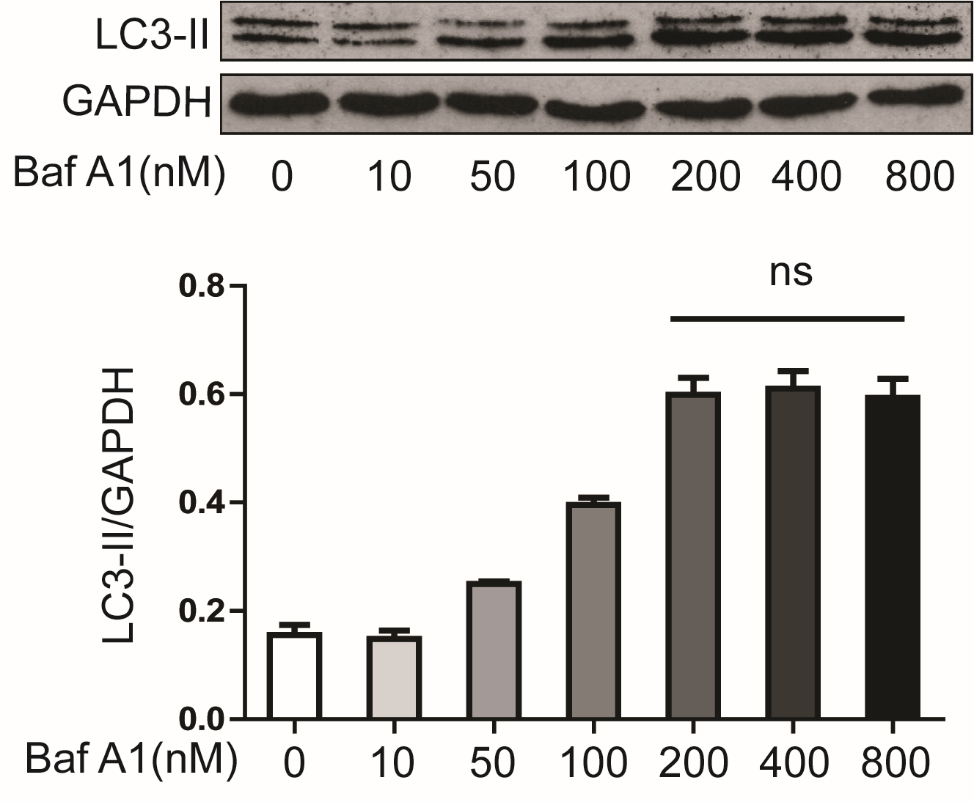
**

**Figure s2.** The response-profile of TCam-2 cells to different concentrations of Baf A1 indicates that 200nM is the best concentration to inhibit lysosomal activity in these cells.(ns, nonsigniﬁcant)
